# Supplementary material for: Patterns of Intron Gain and Loss in Fungi
Source: PLoS Biol. 2004 Nov 30;2(12):e422. doi: 10.1371/journal.pbio.0020422 (PMC532390; doi:10.1371/journal.pbio.0020422)
Supplement: Table S1 — Also available at http://genes.mit.edu/NielsenEtAl/. (4.3 MB ZIP). [file pbio.0020422.st001.zip › NielsenEtAl/html/271.html]

AN5194.1.NCU02544.1.MG01563.1.FG01388.1


```
 CLUSTAL W (1.82) Multiple Sequence Alignments - Introns Inserted


Sequence 1: NCU02544.1	1078 aa
Sequence 2: FG01388.1	1062 aa
Sequence 3: MG01563.1	1079 aa
Sequence 4: AN5194.1	963 aa
Alignment Length: 1103 aa
Number Identitical Residues: 539 aa
Alignment Score (without introns) 27033


MG01563.1 	MPPRLRTALALGATSLLWLASVSAN--YTTYDSMRAQLALMDDRPKDCPPC2FNCLLPAY
NCU02544.1	MAFGIPRALALAALSLSCVASAYQN--YTSIDMLRAQMELMADRPKDCPPC2FNCNLPMY
FG01388.1 	MAPALRSLLIGAVAFWPGIAMASHNATYSQADQLRAQLALMGDRPDGCPPC2FNCLLPAH
AN5194.1  	---------------------------------------------------~--------
          	                                                            

MG01563.1 	TCGQYGGCNEYDGTCRCPEGYGGLDCMEPL1CGSLARGKDRPMRSGDQCSCDEGWTGINC
NCU02544.1	SCGQFASCSEYSGKCICPDGFGGDNCLEPL1CGSLARGPDRLARKGKSCECDDGWTGINC
FG01388.1 	TCAQYAGCNEFNGKCACPEGFGGDDCLEPL1CGSLPKGKDRPMRSGSTCECDEGWTGINC
AN5194.1  	------------------------------~-----------------------------
          	                                                            

MG01563.1 	NVCTENKYCDALMPENEGG~VCYQMGDVVNHNYQMCDVTNKPILDMLGGRKPQVTFDCDK
NCU02544.1	NVCTNDNVCNAFTETGDGG~VCYTGGEVVKQNYQICDVTNKAIRNLLGTKVPQVTFTCKK
FG01388.1 	NVCTDNKACNALMDKGEGG0--------------ICDVTNQKITSLLGKQRPEVTFTCKR
AN5194.1  	------------MPTGEDG~VCYKQGVTVKENFQMCDVTNRKILDQLKERKPQVTFSCEA
          	               .:.*  .   .   . . .:*****: * . *  : *:*** *. 

MG01563.1 	ASGECDFQF1WIDQKESFYCHLNKCDASA--EFDESRNSTNYDCGKVNCSCIPGRFLCGE
NCU02544.1	ETGECDFQF1WVEERESFMCHLRECDSTANFDFDNGKNTTSYKCEKIQCECIPDRMLCGE
FG01388.1 	EDQTCDFQF1WVDAIESFYCHLSECDSTA--DWEESKNTTSYKCNKISCSCIPDRMLCGK
AN5194.1  	EDKTCNFQF~WVDQVESFYCGLDTCDWGL--ETDYDKNTTHYKCENINCKCIPGRMLCGE
          	    *:*** *::  *** * *  **     : : .:*:* *.* ::.*.***.*:***:

MG01563.1 	SGSVDIGDFLDQEIKGPATFKCLQKTGDKNN--CDFDEAAMNDLVSSIFGDKTITLSCRS
NCU02544.1	HGSVDIGDFLSEQIKGPASFECTQTTGEEKK--CAFKEPEMNNLINDVFGDQSILLTCSA
FG01388.1 	DGSIDLTDFLDQSIQGPGRFECTQKHGGTHD--CAFKEPEMDALILALIGDSSIFLDCHA
AN5194.1  	EGSIDIGEFLKQSIKGPATFSSVSTVGGSKEDGSKFQEPEMDNLIKSVFGDESITLKCSS
          	 **:*: :**.:.*:**. *.. .. *  :.... *.*. *: *:  ::**.:* * * :

MG01563.1 	GECLYHTQVPGYKRPVSKINTPLIAGVIAMCSIFVAVTIIIIWYLSRRKFRYGPIHL---
NCU02544.1	GECLYHTEVPGYTPTVPKINTPLIAGVIAACGLFLVAVILGTWYLSRRKFNYGPIHL---
FG01388.1 	GECLYETEVPGWKRPVKQINTPLIAGVIAGCSLFLVAVIILTWYLSRRKLNYGAIRL---
AN5194.1  	GECVHKTEVPGYQRPVKKINTPLIAGVIAGCALFVVAVILGLWYQSRR-SRYSRIQLSLS
          	***::.*:***:  .* :*********** *.:*:...*:  ** ***  .*. *:*: :

MG01563.1 	DDSDDEQVKLMADHKPASLYFENVCYGLNGRQILSEIQGMAHPGEITAIMGASGAGKTTF
NCU02544.1	DDSDDEAIKLMTDHKPASLYFENVVYNLNGKEILSGIQGMAHPGEVTAIMGASGAGKTTF
FG01388.1 	EDSDDESTKLMADHKPASLYFEDVAYNLNGKQILTGIRGICKPGEVTAIMGASGAGKTTF
AN5194.1  	DDSDDEASKLLADHRPAALYWDNVSYFVNGKEILSGIQGVAQPGQITAIMGASGAGKTTF
          	:*****  **::**:**:**:::* * :**::**: *:*:.:**::**************

MG01563.1 	LDILARKNKRGVVGGDFFVNGEKVNDTDYKNVVGFVDQEDTMLPTLTVHETILNSALLRL
NCU02544.1	LDILARKNKRGQVSGDFYINGEKVSDPEYKNAVGFVDQEDTMLPTLTVHETILNSALLRL
FG01388.1 	LDILARKNKRGQVHGNFYVNGEKVDDSDYKNVVGFVDQEDTMLPTLTVHETILTSALLRL
AN5194.1  	LDLLARKNKRGIAQGDFYVNGEKLDDADFKSMIGFVDQEDTMLPTLTVHETILTSALLRL
          	**:******** . *:*::****:.*.::*. :********************.******

MG01563.1 	PREMSRQAKEQRVYEVEKQLGIYHIRDSLIGSEEGKGRGISGGEKRRVGIACELVTSPSI
NCU02544.1	PKDMTRAAKEQRVIEVEKQLGIYHIRDSLIGSEEGKGRGISGGEKRRVGIACELVTSPSI
FG01388.1 	PRDMGRAAKEQRVLEVEKELGIHHIRDSLIGSEEGKGRGISGGEKRRVGIACELVTSPSI
AN5194.1  	PRDMSRAAKEQRVTEVEKQLGIHHIKDQLIGSEEGSGRGISGGEKRRVGIACELVTSPSI
          	*::* * ****** ****:***:**:*.*******.************************

MG01563.1 	LFLDEPTSGLDAYNAYNV~IECLVNLAKTYKRTVIFTIHQPRSNIVALFDRLILLAQGKT
NCU02544.1	LFLDEPTSGLDAYNAYNV~VECLVTLAKTYKRTVIFTIHQPRSNIVALFDRLILLAQGKT
FG01388.1 	LFLDEPTSGLDAYNAYNV~IECLVTLAKTYKRTVIFTIHQPRSNIVALFDRLVLLAQGRT
AN5194.1  	LFLDEPTSGLDAFNAFNV2VECLVSLAKDYNRTVIFTIHQPRSNIVALFDRLILLANGKT
          	************:**:** :****.*** *:*********************:***:*:*

MG01563.1 	VYSGPFHQCQDYFDRIGYACPPGFNIADYLVDLTMHAGTTTSFDDG-------SISADVA
NCU02544.1	VYSGPLHQCQEYFDQIGYTCPPGFNIADYLVDLTMHAGSTSSYDDG-------TLSVDGV
FG01388.1 	VYSGPFNQCQSYFDEIGYECPPGFNIADYLVDLTMHASSTQSVDDG-------TVGLDNS
AN5194.1  	VYSGPFSTCQQYFDDAGYTCPPGFNIADYLVDLTMHAGSTTQFHSDEEESPLLAVRSDPP
          	*****:  **.***  ** ******************.:* . ......:.  ::  *  

MG01563.1 	SVGPSSTRAVKSIASISGGSIGEDSGAEGSSSTRPKNKRRDSVKTRQERELFTRRKN---
NCU02544.1	SVGPSSTRAVKSIASVSGVSIGDDSLVESSSS-RPRNKRRDSVRRRQERELYTRRKQ---
FG01388.1 	SVGPSSTRAIKSVASISGTTSGDD---DAGSS-RPKTHRRHSVKLRQERQLFTRRK----
AN5194.1  	KTASSSLRAVKSVASASNASIEDSSNSFDYGR-RPKGKRRTSLKQRQDRQLYSRRKDADA
          	....** **:**:** *. :  :.:     .  **: :** *:: **:*:*::***.:.:

MG01563.1 	----TVDTAASSDAG-EEIGAYRLQKQPHNTEASQLTADDPHDLPPAALSGTDLDMLVNS
NCU02544.1	----AVDTAASSDAG-DEIGGYKLQKQPP-VTPLRSTNDDLHDLPPLAATGTDLDVLIES
FG01388.1 	----TVDTAASSDAGGEDNGAYRLHQNPV-DSLTAPLLEDPHDLPPAAATGTDLDIMTQS
AN5194.1  	PPTPRTDEEDAAEVGESQQHWLRLSRQQG--QTPPQILDDPHDLPPAASGQTDLDLMVAH
          	..:. .*   :::.* .:    :* ::           :* ***** *   ****::   

MG01563.1 	FQQSDIAGNTHDEIHQAIAGARSANGSNSNGYSTAGPNIHTSVMGRGYARVSYARQFVIL
NCU02544.1	YIHSDIAASTHEEIHQAIAAAVNSNGQNSNGYVADG-NIYTGTMGKGYARVGLFRQFVIL
FG01388.1 	FMHSDIARSTHEEIQQAIQTADNANGTNSNGYTTQGPNIHVSAVGKGYARVGYLRQFIIL
AN5194.1  	YAASDIARNVRNEIISATQRASAGNGS-VNSEVPSSPLVQT---PKSYARVSLARQFIIL
          	:  **** ..::** .*   *  .**   *.  . .. : .    :.****.  ***:**

MG01563.1 	SRRTWINLYRNPMLMLMHYAMAILLGVLSGFLFYGLTSDIAGFQNRLGLFFFILALFGFS
NCU02544.1	SQRTWKNLYRNPMLMLTHYAIAILLAVFAGYLFYGLTLDIAGFQNRLGLFFFVLALFGFS
FG01388.1 	SQRTWKNLYRNPMLMLTHYAIAIVLAVFSGYLFYGLTDDIPGFQNRLGLFFFLLALFGFS
AN5194.1  	SQRTWRNLHRNPMLMLTHYAIAILLAVLCGFLFYGLTDDIKGFQNRLGLFFFVLALFGFS
          	*:*** **:******* ***:**:*.*:.*:****** ** ***********:*******

MG01563.1 	TLTSLAVFSSERLLFVKERANGYYSPITYFAAKVIFDIIPLRIIPPILMGSIIYPMTGLV
NCU02544.1	TLTSLGVFSQERLLFVRERANGYYSPITYFAAKVLFDIVPLRIIPPILLGAIIYPMTGLV
FG01388.1 	TLTSLNVFATERLLFVRERANGYYAPATYFAAKVLFDIIPLRIIPPILMGSIIYPMTGLV
AN5194.1  	TLTSLTVFSTERLLFVRERANGYYHPVTYYAAKVVFDIVPLRLIPPIIMGVIVYPMTGLI
          	***** **: ******:******* * **:****:***:***:****::* *:******:

MG01563.1 	PDAPHYFTFITVLVLFNLAAAAICLFLGIVCKDGSVANLLGSLVMLFSLLFAGLLLNQNA
NCU02544.1	ADYQRFFVFILVLVLFNLAAAAICLFIGILCKDGGVANLIGSLVMLFSLLFAGLLLNHNA
FG01388.1 	ADSTHFFNFILVLVLFNMAAAAVCLFIGIVCKDGSVANLIGSLVMLFSLLFAGFLLNHKA
AN5194.1  	PAWPEFFKFILVLVLFNLAAATICLFIGIVFRDGGVANLIGSLVMLFSLLFAGLLLNHDA
          	.   .:* ** ******:***::***:**: :**.****:*************:***:.*

MG01563.1 	IPDPAKWLQS~LSIFHYGFESLIVNEVSHLQLIDNKYGLDITVPGASILSSFGFNNNALW
NCU02544.1	IPAAALWLQW~LSIFHYGFEALIVNEVTQLTLVDHKYGLDITVPGATILTTFGFDNSAMW
FG01388.1 	TPKGALWLQT~LSIFHYGFESLIVNEVLHLTLVDSKYGLDITVPGAAILSSFGFNNDAMW
AN5194.1  	IPPSALWLQS0LSIFHYGFEALIVNEVTFLTLIDHKYGLDIEVPGASILSAFGFDTLAYW
          	 *  * ***  *********:******  * *:* ****** ****:**::***:. * *

MG01563.1 	PDVINLGIFGVMFIILAYLAMHFLLVERR
NCU02544.1	RDIMNLGLFGMTFIILAYGAMHILLVEKR
FG01388.1 	PDIRNLGIFAAVFIVLSYAAMHILLVERR
AN5194.1  	KDVIGLAVFSGAFLVIAYIAMHFLLVERR
          	 *: .*.:*.  *::::* ***:****:*
```
